# Thymic epithelial cell-derived signals control B progenitor formation and proliferation in the thymus by regulating *Let-7* and *Arid3a*

**DOI:** 10.1371/journal.pone.0193188

**Published:** 2018-02-20

**Authors:** Shiyun Xiao, Wen Zhang, Nancy R. Manley

**Affiliations:** Department of Genetics, Paul D. Coverdell Center, University of Georgia, Athens, Georgia, United States of America; RIKEN Center for Integrative Medical Science, JAPAN

## Abstract

The postnatal thymus is an efficient microenvironment for T cell specification and differentiation. B cells are also present in the thymus and have been recently shown to impact T cell selection, however, the mechanisms controlling B cell development in the thymus are largely unknown. In *Foxn1*^*lacZ*^ mutant mice, down-regulation of *Foxn1* expression in thymic epithelial cells beginning 1 week after birth caused a dramatic reduction of T progenitors and an increase of B cell progenitors. This time point is coincident with the switch from fetal to adult-type hematopoietic stem cells (HSCs), which is regulated by the *Lin28-Let7* system. We hypothesize that the thymic environment might regulate this process to suppress fetal-type B cell development in the thymus. In this study we show that in the *Foxn1*^*lacZ*^ thymus, although the down-regulation of *Lin28* in thymocytes was normal, up-regulation of *Let-7* was impaired. The failure to up-regulate *Let-7* caused a transient increase of *Arid3a* in B precursors, which is known to promote fetal-type B cell fate. Over-expression of *Lin28a* in HSCs also reduced *Let-7* and promoted *Arid3a* expression in BM and thymic B progenitors, increasing B cell production in the thymus. The level of *Let-7* in thymic B progenitors was up regulated by *in vitro* co-culture with IL15, Vitamin-D3, and retinoic acid, thus down-regulating *Arid3a* to promote B cell differentiation. All of these signals were produced in thymic epithelial cells (TECs) related to Let-7 expression in thymic B progenitors, and down-regulated in *Foxn1*^*lacZ*^ mutants. Our data show that signals provided by TEC control thymic B cell development by up-regulating *Let-7*, suppressing *Arid3a* expression in intrathymic progenitor B cells to limit their proliferation during the neonatal to adult transition.

## Introduction

Hematopoietic stem cells (HSCs) undergo a developmental program change during ontogeny including changes in hematopoiesis sites, self-renewal activities, gene expression profiles, lineage biases, and differential intrinsic properties and differentiation potentials [1–3]. Two distinguishable properties of HSCs have been defined as specific characteristics of fetal (FL-HSCs) and adult (BM-HSCs) [3,4]. The switch from fetal to adult type HSC profiles has been proposed to occur in the period between one to three weeks after birth [3–5]. Fetal and adult HSC types have also been shown to have different potential for differentiation in the thymus. For example, Vγ5^+^ γδ T cells can only be generated from FL-HSCs in fetal thymus, but not from BM-HSCs [6,7]. Also, IL7 is required for adult thymocyte development but not for the production of thymocytes during fetal thymopoiesis [8,9]. However, the total range of effects due to the switch of HSCs from fetal to adult type on the thymocytes development, and the cell autonomous and non-autonomous mechanisms controlling these differences, remain open questions.

The Lin28b/Let-7 microRNA (miRNA) system plays a critical role in the distinct differentiation potential of fetal and adult derived HSCs in both mice and humans [5,10]. *Lin28b* is expressed in FL derived precursors and newborn (NB) thymocytes, but is dramatically reduced one week later in postnatal thymocytes and is absent in adult BM precursors. Conversely, *Let-7* is highly expressed in adult BM but is very low in FL precursors [5,10]. Ectopic expression of *Lin28b* in adult BM or *Let-7* in FL precursors is sufficient to switch these precursors to a reversed developmental pathway in B cell development [5,11]. The redirection of fetal to adult-type switch may occur after B cell commitment at the Pro-B stage, and the switch from fetal to adult-type HSCs occurs around one week after birth [5,11].

ARID3a (AT-rich interaction domain, also called Bright) was firstly characterized as a key transcription factor associated with the increase of transcription of the immunoglobulin heavy chain locus in activated B cells [12,13]. *Arid3a* is highly expressed in progenitor B cells including pro-B and pre-B cells but not IgM^+^ immature B cells in BM, and its expression is tightly regulated at the level of transcription throughout B cell differentiation [12,13]. A recent study showed that the Arid3a mRNA contains several Let-7s target sites, and that its gene expression can be induced by Lin28b and repressed by Let-7s. Retroviral transduction of Arid3a in adult BM pro-B cells is sufficient to switch B cell development from adult-type to fetal-type B cells. Conversely, silencing of Arid3a by retroviral shRNA transduction in fetal pro-B cells could redirect the fetal cells to adult-type B cell development. Thus, Arid3a is a key transcription factor regulated by the Lin28b-Let-7 system that controls the B cell developmental switch between fetal and adult-type [11]. In addition, *Let-7* in hematopoietic cells regulates the differentiation of NKT cells in the thymus. This effect is critically mediated by signals derived from the thymic microenvironment [14]. Thus, we hypothesize that the thymic microenvironment might also regulate *Let-7* expression in thymic B cells to control their development, in particular during the B cell precursor switch from fetal to adult type in the thymus during the neonatal to young adult transition.

We previously generated a novel *Foxn1* allele, designated *Foxn1*^*lacZ*^ [15]. In this model, expression of the critical TEC-specific transcription factor *Foxn1* is normal at fetal stages, but down-regulated beginning at postnatal day 7, causing progressive reduction of total thymocytes and premature thymic involution. In the accompanying paper, we have shown that immediately after *Foxn1* down-regulation in these mutants, there was both a decrease in T lineage-committed DN1a/b cells and a transient increase in intrathymic B progenitors. We also provided evidence that although the lineage change of thymic seeding progenitors (TSPs) toward B cell development correlated to changes in the thymic microenvironment including *Dll4* and *IL-7* signals, the timing of thymic B cell production was not consistent with that of defects in the thymic microenvironment. These data suggest that the critical factor for thymic B development is a cell-intrinsic change in B potential of HSCs during the neonatal to young adult transition (companion manuscript submitted to PLoS One). Postnatal day 7 is a key time point for both the switch from fetal to adult HSC phenotypes[3–5], and for the change in B potential of TSPs [16,17]. Based on the timing of this T, B lineage change at day 7, we further hypothesize that the B potential change in TSPs is mechanistically related to the switch from fetal to adult type HSCs. In this study, we show that signals from TECs in the thymic microenvironment regulate the *Lin28/Let-7* axis, thus suppressing *Arid3a* expression within hematopoietic-derived cells to control B cell differentiation. Furthermore, these effects occur specifically at thymic pro-B and pre-B stages, and regulate both proliferation and differentiation.

## Material and methods

### Mice

*Foxn1*^*lacZ/lacZ*^ (Z/Z) mice were generated as described previously on a C57Bl6/J background [15,18]. *Foxn1*^*nude*^ heterozygous (*Foxn1*^*+/nude*^*)* mice on a C57Bl6/J background were purchased from The Jackson Laboratory (Bar Harbor, ME). *Foxn1*^*lacZ/nude*^ (Z/N) mice were generated by crossing Z/Z with *Foxn1*^*+/nude*^ mice. *iLin28a* inducible transgenic mice were originally provided by Eric Moss (Department of Molecular Biology, Rowan University, Stratford, NJ 08084 USA) and transferred from Dr. Jianfu Chen (Department of Genetics, University of Georgia) [19]. *Vav-iCre* mice were purchased from The Jackson Laboratory [20]. All analysis was performed on control and mutant or transgenic animals from within the same interbreeding colony, using littermate animals whenever possible. *Foxn1*^*+/lacZ*^ (+/Z) mice were used as controls for the *Z*/Z and *Z/nu* mice, based on our previously published data [15,18] and on our current analysis showing that B cell phenotypes and frequencies are similar in wild-type and *+/Z* mice (S1 Fig). All mice were maintained in a specific pathogen-free facility at the University of Georgia. The experiments were approved by the University of Georgia Institutional Animal Care and Use Committee.

### Flow cytometry

Freshly isolated thymocytes in suspension (1×10^6^) were used for each sample. Cells were blocked by anti-CD16/32 (Clone:93) antibody before staining. Total thymic cells gated on CD19^+^B220^+^ (6D5, RA3-6B2) were analyzed for CD43 (1B11), CD24-FITC (M1/69), IgM (RMM-1), IgD (11-26c.2a) or CD5 (53–7.3) in a different panel. For NKT cells, total thymocytes were stained by PerCp labeled anti-CD4 (GK1.5), CD8 (53–6.7), CD25 (3C7), anti-CD44 APC (IM7), anti-TCRβ (H57-597) and anti-KN1.1 (PK136) and then gated on CD4^-^CD8^-^CD25^-^CD44^+^ and TCRβ^+^ DN1 to show the profile of NK1.1 and CD44 expression. All antibodies were purchased from Biolegend (San Diego, CA). The data were analyzed using Flowjo^™^ software.

### Cell sorting

For TEC sorting, thymic lobes were cut into 1 mm^3^ pieces and gently washed in 2% FBS + RPMI1640 medium to partially remove thymocytes. The thymic pieces were then transferred to a digestion solution containing collagenase/dispose (Roche) at 1mg/ml and DNase I 20ng/ml in 8ml of 2% FBS + PRMI1640 medium, and then placed into a 37°C water bath to digest for 60 minutes. The thymic tissue was gently agitated by plastic pipette every 20 minutes. The cells were filtered by passing through a size 70μm cell strainer and then incubated with anti-CD45 APC (30-F11), anti-EpCam PE (G8.8) and anti-MHCII FITC (M5/114.15.2). TECs were sorted as CD45^-^Epcam^+^MHCII^+^ cells. For medullary and cortical TECs cell sorting, UEA-1^+^ TECs were sorted as mTECs and UEA-1^-^ TECs as cTECs using Biotin-UEA-1 following avidin-APC-Cy7.

For thymic B cell sorting, a total thymus suspension was passed through a cell strainer, and cells were stained by CD19 PE-Cy7, B220 APC, IgM + Lin PE, CD24 FITC and CD43 APC-Cy7 (1B11). Thymic progenitor B cells were gated on CD19^+^B220^+^CD24^+^CD43^+/lo^IgM^-^ subpopulations. All cells were sorted using a MoFlo^™^ cell sorter.

### BrdU incorporation

Each mouse was injected with 1 mg of BrdU (Sigma-Aldrich) once i.p. and then fed with BrdU-containing water (0.8 mg/ml) for three days. The thymocytes were harvested for CD4, CD8, CD25, CD44 and CD19 surface-staining, fixed and permeabilized in PBS containing 1% paraformaldehyde plus 0.01% Tween20 for 48 h at 4°C, and then submitted to the BrdU DNaseI detection technique. FITC-anti-BrdU (clone 3D4; BD Pharmingen) was used for BrdU staining.

### RT-PCR and Q-PCR

mRNA from sorted TEC subsets and B cells were extracted by RNeasy micro kit (GIAGEN). Gene expressions of *Ccl25*, *Aire*, *IL15*, *Cyp27b1*, *Aldh1a2* in subsets of TECs, and *Lin28b*, *Let-7g*, *Let-7b* and *Arid3a* in fresh progenitor B cells and cultured thymic B cells were measured by Q-PCR, with a *GAPDH* FAM primer/probe used as an endogenous control. All primers/probes were ordered from Applied Biosystems. Q-PCR was performed following the instruction of the manufacturer in 10μl volume using the AB 7500 Sequence Detector.

### Cell culture

The major population of thymic progenitor B cells defined as B220^+^CD19^+^CD43^+/lo^CD24^+^IgM^-^ was sorted from Z/Z and *Z/N* mutant thymus. 1 × 10^5^ sorted cells per well were seeded into 96 well plates in present of IL15 (50ng/ml; R&D Systems), 1α,25-dihydroxy vitamin D3 (VD3, 100nM; Sigma-Aldrich) and/or all-trans retinoic acid (RA, 100nM; Sigma-Aldrich) alone or in combination. The cell number, phenotype and/or gene expression of cultured B cells were analyzed at 24 and 48hr after cell culture.

### Statistical analysis

All data were collected in a Microsoft Excel file and analyzed using Prism software by student’s t-test, P value in two-Tailed and one-way analysis of variance (ANOVA)-Bonferroni test.

## Results

### Thymic B cells retained a more fetal-like profile in Foxn1^lacz^ mutants

**Fig 1:**
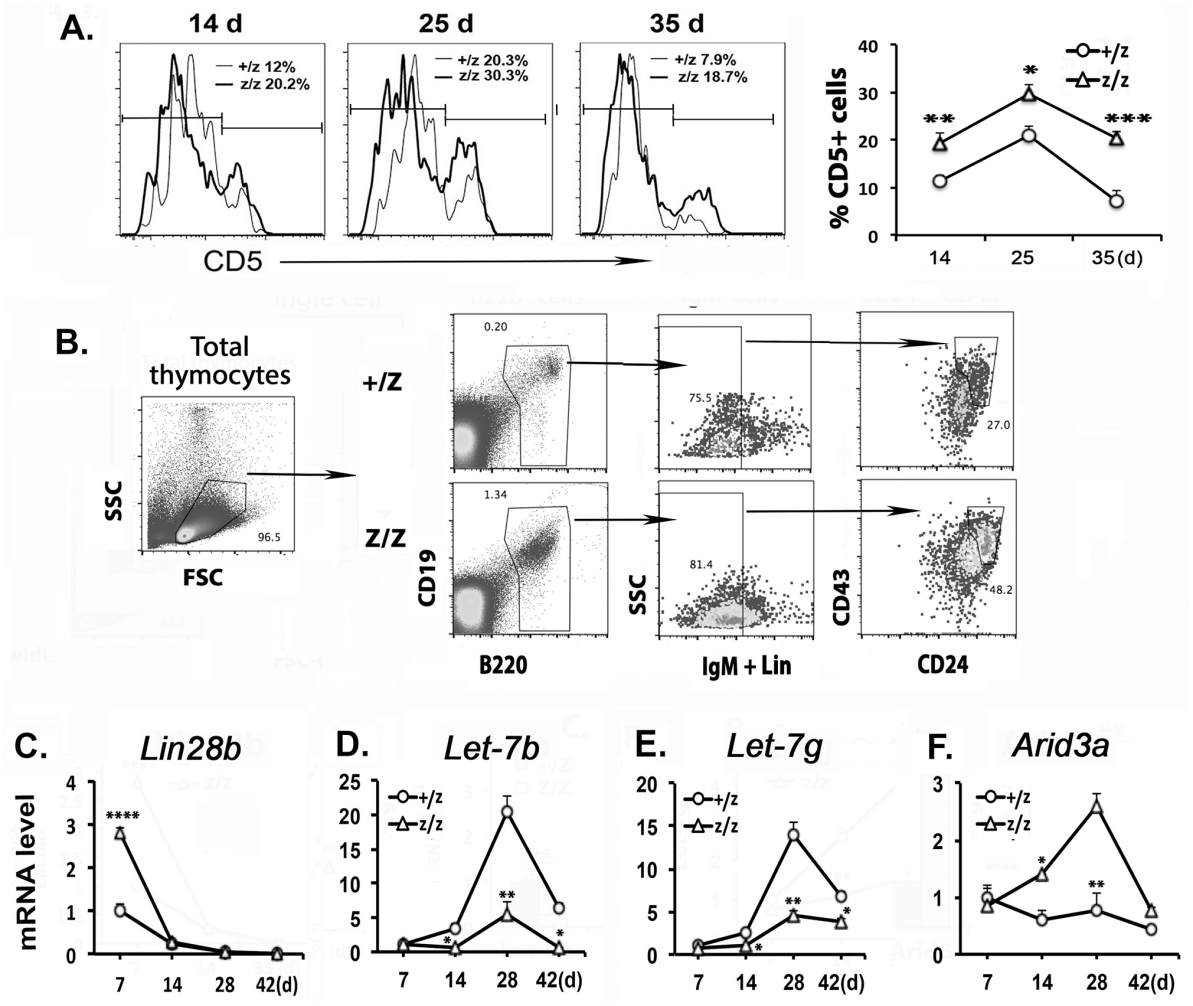
The thymic B progenitors showed an increased fetal-like profile in the *Foxn1*^*lacZ*^ thymus. **(A)**. Overlap of CD5 expression on gated B220^+^CD19^+^ thymic B cells at age of day 14, 25 and 35 in +/Z and Z/Z mice, the summary data was shown on right. **(B)**. The thymic B progenitors were sorted on CD19^+^B220^+^CD24^+^CD43^+/lo^IgM^-^. **(C-F)**. Kinetic gene expressions of *Lin28b* (C) and *Let-7b* (D) *Let-7g* (E) and *Arid3a* (F) from day 7 to 42 in sorted thymic progenitor B cells. Each time point was represented at least five individuals in two independent experiments. Student’s t-test analysis between +/Z and Z/Z (E): *P <0.05, **P <0.01, ***P <0.001, ****P<0.0001. Bars indicate means ± SEM.

To test whether the thymic microenvironment controls the lineage potential of TSPs during the fetal to adult HSC phenotypic switch, we analyzed whether the increased B cells in the Z/Z thymus in the perinatal period were of the B-1a (typical of fetal B progenitors) or B-2 (typical of adult progenitors) type. Phenotypic analysis showed that CD5 expression was significantly increased in Z/Z mutant B cells (Fig 1A), supporting an increased fetal type B progenitor in the *Foxn1*^*lacZ*^ mutant thymus. To test if the *Lin28b/Let-7* system and *Arid3a* expression were changed in these thymic B progenitors in *Foxn1*^*lacZ*^ mutants, we sorted the major thymic progenitor B populations (CD19^+^B220^+^ CD24^+/hi^ CD43^+/lo^IgM^-^) from both +/Z and Z/Z thymus (Fig 1B) and measured these genes’ expression over this time period by Q-PCR. *Lin28b* expression in B progenitors was 2.8-fold higher at day 7 in the Z/Z mutants than in +/Z controls but returned to control levels by day 14, and no *Lin28b* expression was detected at later stages in either genotype (Fig 1C). This down regulation allowed for the normal up-regulation of *Let-7b and Let-7g* in +/Z control B progenitors, peaking at day 28 (Fig 1D). However, *Let-7b* and *Let-7g* expression failed to be up-regulated, remaining more than 3-fold lower in Z/Z B progenitors relative to controls at day 28, and the returning to undetectable levels (Fig 1E). Consistent with these data, *Arid3a* expression (which is normally suppressed by *Let-7*) was increased, peaking at day 28 in Z/Z B progenitors at 3.4-fold higher than in controls (Fig 1F). These results indicated that the progenitor B cells in *Foxn1*^*lacZ*^ thymus retained a more fetal like profile compared to the same cells in +/Z controls. As these changes occurred during the key time period when HSCs switch from fetal to adult-type, we suggest that the defective thymic microenvironment delays the switch of TSPs from fetal to adult-type in the *Foxn1*^*lacZ*^ thymus during the neonatal period.

### Repressed differentiation and increased proliferation of B progenitors in the Foxn1^lacZ^ thymus

**Fig 2:**
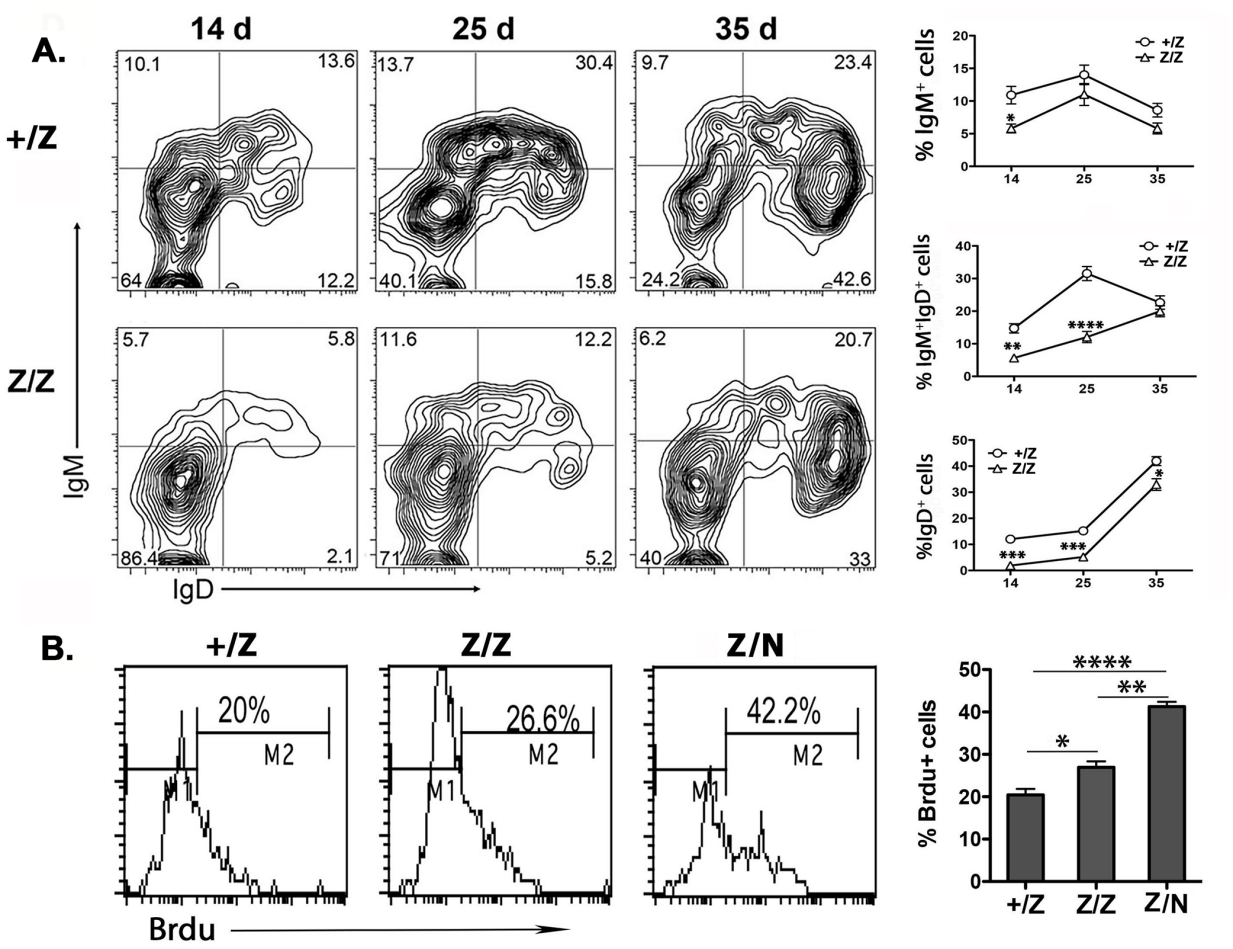
The development of thymic B cells was delayed in the *Foxn1*^*lacZ*^ thymus. **(A)**. The kinetic expressions of IgM and IgD on gated B220^+^CD19^+^ thymic B cells were shown at age of day 14, 25, and 35 in +/Z and Z/Z mice. The summaries of kinetic percentage change of B subsets were shown on the right. **(B)**. BrdU intracellular staining profile in thymic B cells and summary data in +/Z, Z/Z, and Z/N at day 30 mice. Each assay and time point was represented at least five individuals. Student’s t-test analysis for panel A and One-way ANOVA for panel B between +/Z and Z/Z or Z/N: *P <0.05, **P <0.01, ***P <0.001, ****P<0.0001. Bars indicate means ± SEM.

Since our phenotypic analysis identified that most thymic B cells in the *Foxn1*^*lacz*^ thymus have a low level of CD19 and B220, increased expression of Ly51, CD93, and are IgM negative (companion manuscript submitted to PLoS One), we concluded that these increased thymic B cells in the *Foxn1*^*lacZ*^ thymus are similar to the pre-B-II cells at Fr C-D stage in the BM [21]. To further test the effects on the differentiation and/or maturation of these thymic B progenitors, we analyzed the expression of IgM and IgD in these cells over the first postnatal month. Our data showed a delayed expression of IgM and IgD in B cells from *Z/Z* mutant thymus compared to those from +/Z controls (Fig 2A), consistent with the increase of Fr C-D pre B II cells in *the Foxn1*^*lacZ*^ thymus. Conversely, their proliferation was increased, as assessed by BrdU uptake (Fig 2B). These results indicated that these fetal-like B progenitors not only delayed the differentiation and maturation of thymic B progenitors from pre-B to IgM^+^ immature stage, but also promoted the proliferation of progenitor B cells at pre B II stage.

### Suppression of Let-7 activity in HSCs promoted the production of thymic B cells in the Z/Z mutant thymus

**Fig 3:**
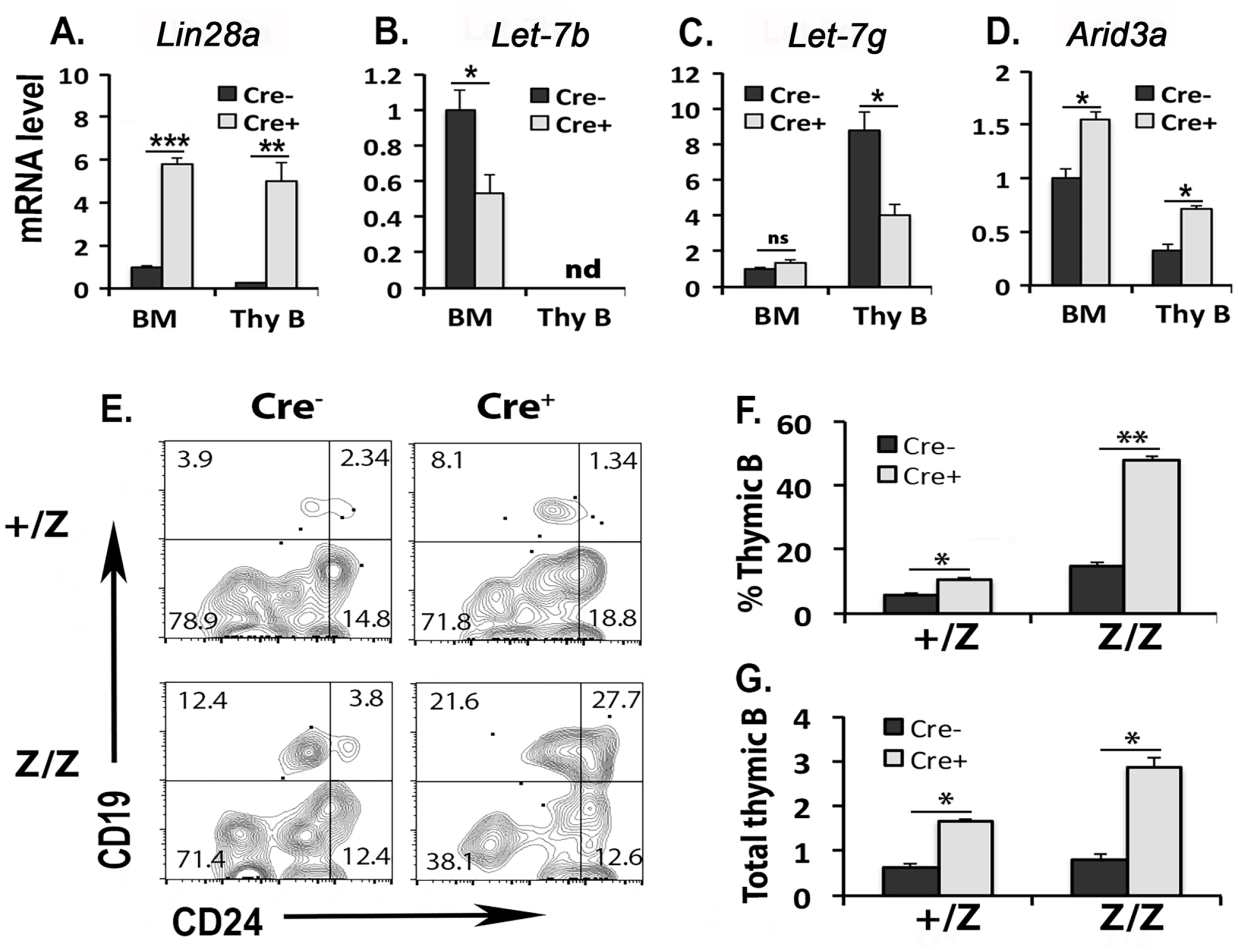
Specific overexpression of Lin28a in HSCs caused a reduction of *Let-7* and promoted thymic B cells production. 1×10^7^ T/ B depleted BM cells isolated from 9-weeks *iLin28a;Vav-iCre* mice including *Cre*^+^ test and *Cre*^-^ control group were transferred into lethally irradiated 8-weeks +/Z and Z/Z mutant mice respectively. The BM and thymic B progenitors (CD19^+^B220^+^CD24^+^CD43^+/lo^IgM^-^) collected from host were analyzed at 21 days later. **(A-D)**. Gene expression of *Lin28a* (A), *Let-7b* (B), *Let-7g* (C) and *Arid3a* (D) in host BM and thymic B progenitors were analyzed by Q-PCR. The value of gene expression in *Cre*^-^ control BM cell was set as 1. Data are representative of three independent experiments (BM: n = 3, thymic B: n = 5 for each group). **(E)**. Profiles of CD19 and CD24 expressions were shown in DN1 thymocytes. **(F-G)**. Percentages of CD19^+^ cells in DN1 thymocytes (F) and a total number of CD19^+^ cells in the thymus (G) were summarized in the histogram. Data are representative of three independent experiments (+/Z: *Cre*^-^ n = 3, *Cre*^+^ n = 3; Z/Z: *Cre*^-^ n = 3, *Cre*^+^ n = 5). ns: not significant. nd: not detected. Student’s t-test results between *Cre*^+^ test and *Cre*^-^ control groups: *P <0.05, **P <0.01, ***P <0.001. Bars indicate means ± SEM.

To test whether the failure to up-regulate *Let-7* in TSPs in the *Foxn1*^*lacz*^ mutant thymus would be sufficient to cause these thymic B cell phenotypes, we generated HSC-specific Lin28a overexpression by crossing the *iLin28a* transgenic mice [19] with *Vav-iCre* [20]. Overexpression of Lin28a in HSCs by this approach has been shown to prevent up-regulation of *Let-7* and block the transition from fetal to adult HSC, reprogramming adult HSCs to a persistent fetal HSC phenotype [5,10]. We transferred T, B deleted BM cells from 9-week-old *iLin28a;Vav-iCre* mice (well after the age when B cell potential has declined) into lethally irradiated 8-week-old *Foxn1*^*lacz*^ mutant and control mice to see if enforced suppression of *Let-7* would be sufficient to maintain a high B potential in TSPs in the thymus. Expression of *Lin28a* in both BM and thymic CD19^+^B220^+^CD24^+^CD43^+/lo^IgM^-^ B progenitors was around 5-fold higher in *Cre*^+^ mice than in *Cre*^-^ controls (Fig 3A). Compared to *Cre*^-^ controls, *Let-7b* was reduced 50% in BM cells and not detected in thymic B progenitors; *Let-7g* was more than 50% reduced in thymic B progenitors (Fig 3B and 3C). Consistent with this reduction in *Let-7*, *Arid3a* expression was significantly increased in both BM and thymic B progenitors (Fig 3D). More than half of the thymic B progenitors in *Cre*^+^ 9-weeks-old BM cells group were of the CD24^hi^ pre-B phenotype in Z/Z thymus (Fig 3E and 3F). The total number of thymic B cells was also significantly increased by 2-fold in +/Z, and 3-fold in Z/Z thymus (Fig 3G). These results indicate that reduction of *Let-7* in HSCs is sufficient to cause a higher potential for thymic B production, consistent with our conclusion that failure to up-regulate *Let-7* and the subsequent increase of *Arid3a* are critical factors causing thymic progenitor B cell development in the *Foxn1*^*lacz*^ mutant thymus.

### The signals required for up-regulation of Let-7 in hematopoietic progenitors were reduced in Foxn1^lacZ^ mutant TECs

**Fig 4:**
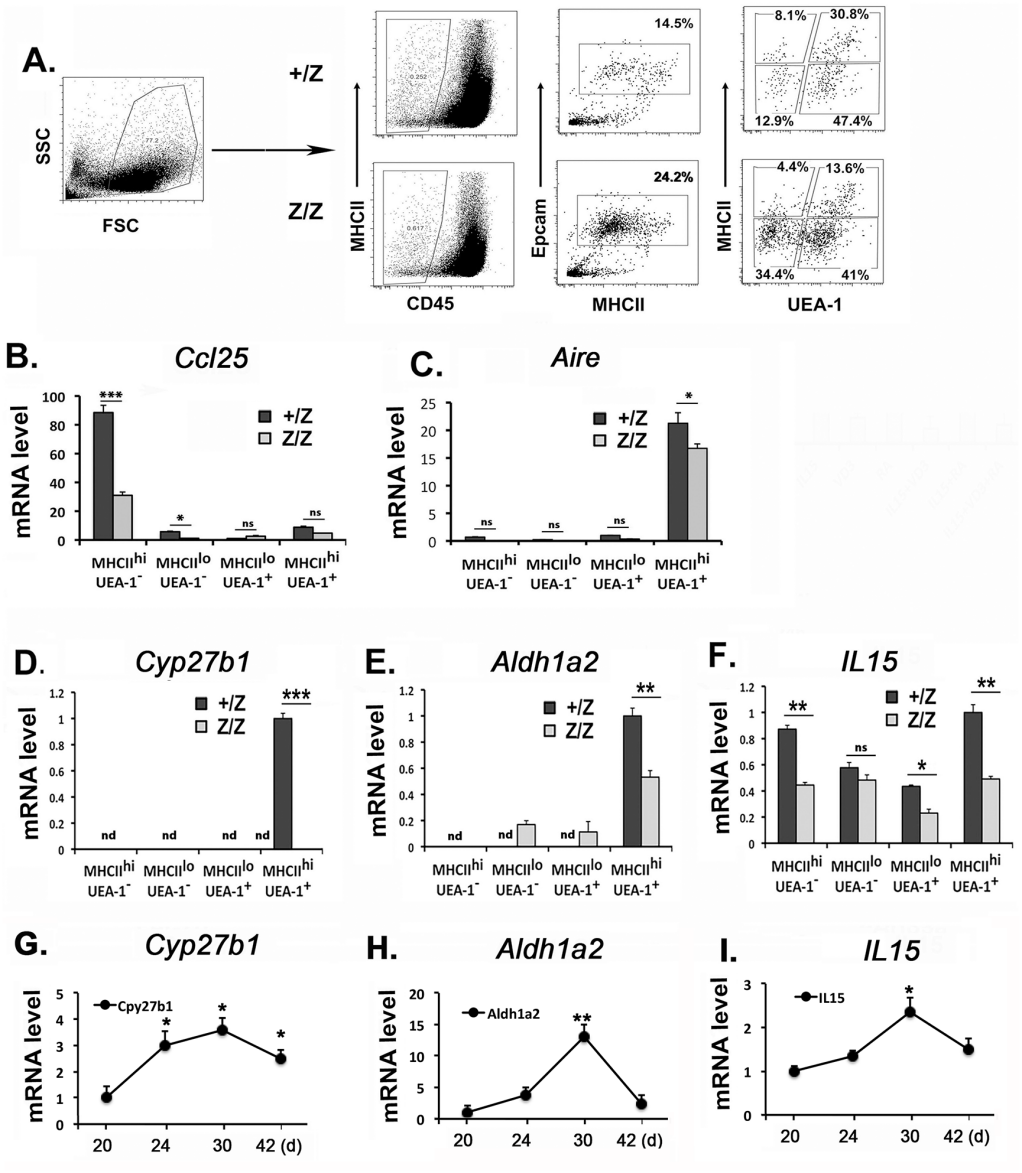
The signals required for up-regulation of *Let-7* in hematopoietic progenitors were reduced from TECs in *Foxn1*^*lacZ*^ mutant. **(A)**. The TECs subsets were sorted by CD45^-^MHCII^+^Epcam^+^ and then separated as MHC^lo^ and MHCII^hi^, cTEC and mTEC four subpopulations by UEA1 staining. Day 30 of +/Z and Z/Z mice were used. (B-F). Gene expressions of *Ccl25* (B), *Aire* (C), *Cyp27b1* (D), *Aldh1a2* (E) and *IL-15* (F) in the TEC subpopulations were then analyzed by Q-PCR. Data are representative of two independent experiments, (+/Z: n = 4, Z/Z: n = 6). nd: not detected. (G-I). The kinetic gene expression of *Cyp27b1* (G), *Aldh1a2* (H) and *IL-15* (I) in MHCII^hi^ TEC subset from 20, 24, 30, and 42 days were analyzed by Q-PCR. Each time point represents at least three individuals in two independent experiments. Data (B-F) Student’s t-test analysis between +/Z and Z/Z. (G-I) One-way ANOVA between day 20 and individual time points. *P <0.05, **P <0.01, ***P <0.001. Bars indicate means ± SEM.

Down regulation of *Lin28b* is necessary for *Let-7g* up-regulation in adult HSCs [22,23]. However, in *Foxn1*^*lacZ*^ mutant thymus, although *Lin28b* was down regulated normally after day 14, both *Let-7b* and *Let-7g* expressions remained low (Fig 1). Up-regulation of *Let-7* in thymic NKT cells has been shown to require IL15, 1α,25-dihydroxy vitamin D3 (VD3) and all-trans retinoic acid (RA) signals provided by mTECs [14]. Since all of these signaling pathways can also affect B cell differentiation [24–31], we considered that these signaling pathways might also regulate *Let-7* expression in thymic B progenitors. To test if these signals were affected by down-regulation of *Foxn1* expression [15], we measured their expression in sorted TEC subpopulations from day 30 +/Z and Z/Z thymus (Fig 4A). As expected, *Ccl25* and *Aire* were highly expressed in sorted MHCII^hi^ cTECs and mTECs respectively (Fig 4B and 4C). The expression of *Cyp27b1* (which produces the active form of VD3), *Aldh1a2* (which produces the active form of RA) and *IL15* were all reduced or absent in Z/Z MHCII^hi^ mTECs (Fig 4D–4F). Further, to test if the down-regulation of Let-7s expression in thymic B cells in the normal thymus after day 30 was due to a reduction of these signals (Fig 1D and 1E), we measured the kinetic expression of these genes over this time period by sorting MHCII^hi^ TEC subsets from wt mice. Consistent with the timing of down-regulation of Let-7s in thymic B cells, the expression of all of these factors peaked at day 30 and then were down regulated at day 42 in MHCII^hi^ TECs (Fig 4G and 4H). These results indicate that these signals produced by TECs normally regulate *Let-7s* expression in thymic B cells during early postnatal thymus development in a temporally dynamic manner.

### The deficiency of stromal-derived signals caused a developmental defect of NKT cells in the Foxn1^lacZ^ mutant thymus

**Fig 5:**
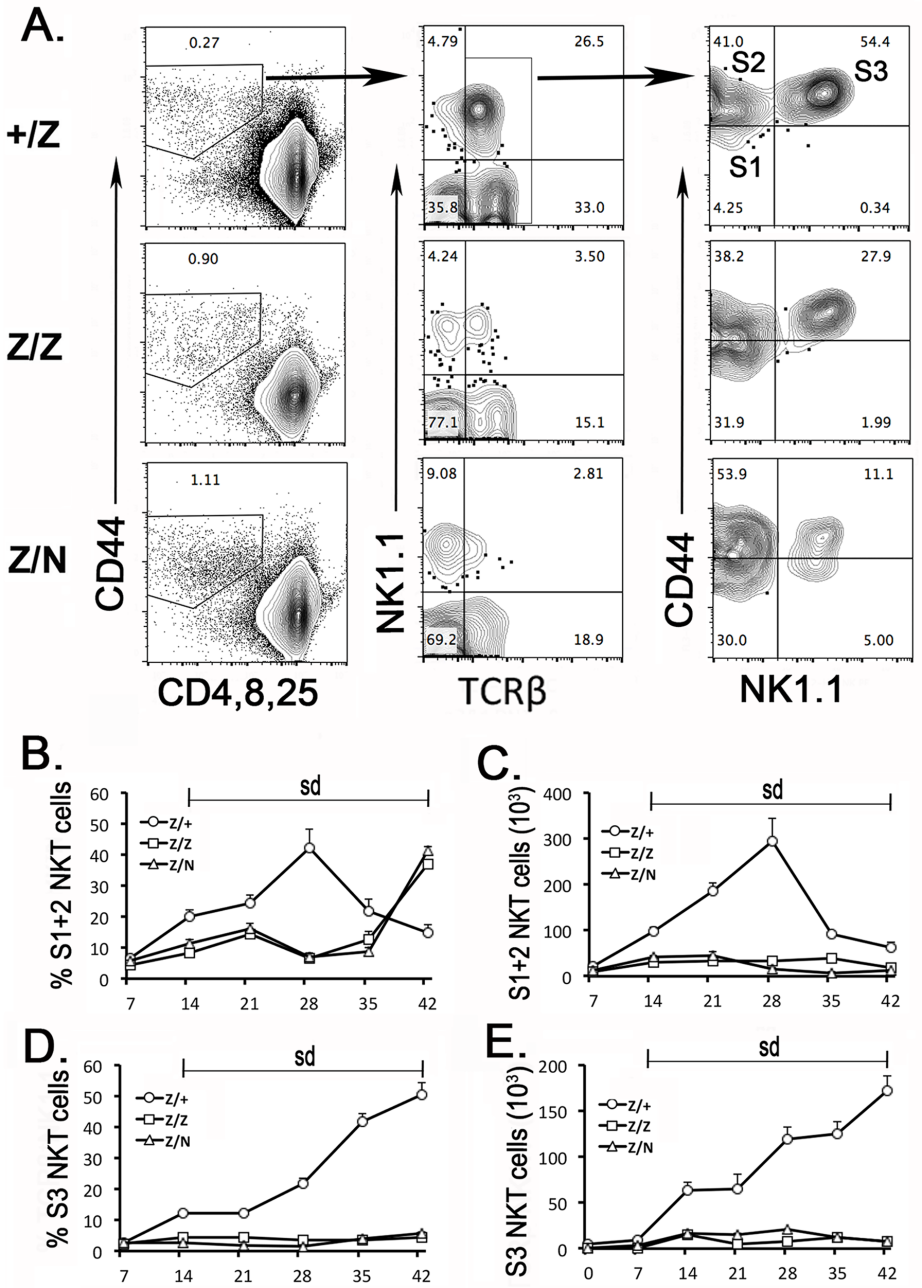
Developmental deficiency of NKT cells in *the Foxn1*^*lacz*^ mutant thymus. **(A)**. Thymic NKT cells were gated on CD44^+^TCRαβ^lo^ DN1 cells. The developmental stage1, 2, 3 of NKT cells were shown by the expression of KN1.1 and CD44 in +/Z, Z/Z and Z/N mice. **(B-C)**. Percentage of immature (S1+S2) (B) and mature (S3) (C) NKT cells. **(D-E)**. Total number of S1+S2 (D) and S3 (E) NKT cells. Each assay and time point was represented at least five individuals. One-way ANOVA between +/Z and Z/Z or Z/N. sd = significant difference, the P value <0.05 in each time point. Bars indicate means ± SEM.

To test the effects of reduction signals of *Cyp27b1*, *Aldh1a2* and *IL-15* on NKT development [14,32] in the *Foxn1*^*lacZ*^ mutant thymus, the DN1 population (CD44^+^CD25^-^ DN) was gated (Fig 5A left panel) to show the profile of TCRαβ expression. The TCRαβ^lo^ DN cells included all stages of NKT cells were significantly reduced in *Foxn1*^*lacZ*^ mutant thymocytes (Fig 5A meddle panel). By gating on TCRαβ^lo^ DN cells, the CD44 and NK1.1 expression profiles showed that the major reduction of cells was CD44^hi^NK1.1^+^ mature NKT (S3) cells (Fig 5A right panel); further kinetic analysis showed a great reduction of NKT cells including immature (S1+S2) and mature (S3) cells in *Foxn1*^*lacz*^ mutant thymus (Fig 5B–5E). These data showed that the differentiation of NKT cells from immature (S1+S2) to mature stage was blocked in *Foxn1*^*lacz*^ mutant thymus, consistent with previous reports [14,32] that down regulation of the *Let-7* regulatory signals in TECs caused a dramatic decrease in NKT thymocytes by blocking terminal maturation of NKT in the thymus.

### Impaired up-regulation of Let-7 in thymic B progenitors was due to the deficient microenvironment in the Foxn1^lacZ^ mutant thymus

**Fig 6:**
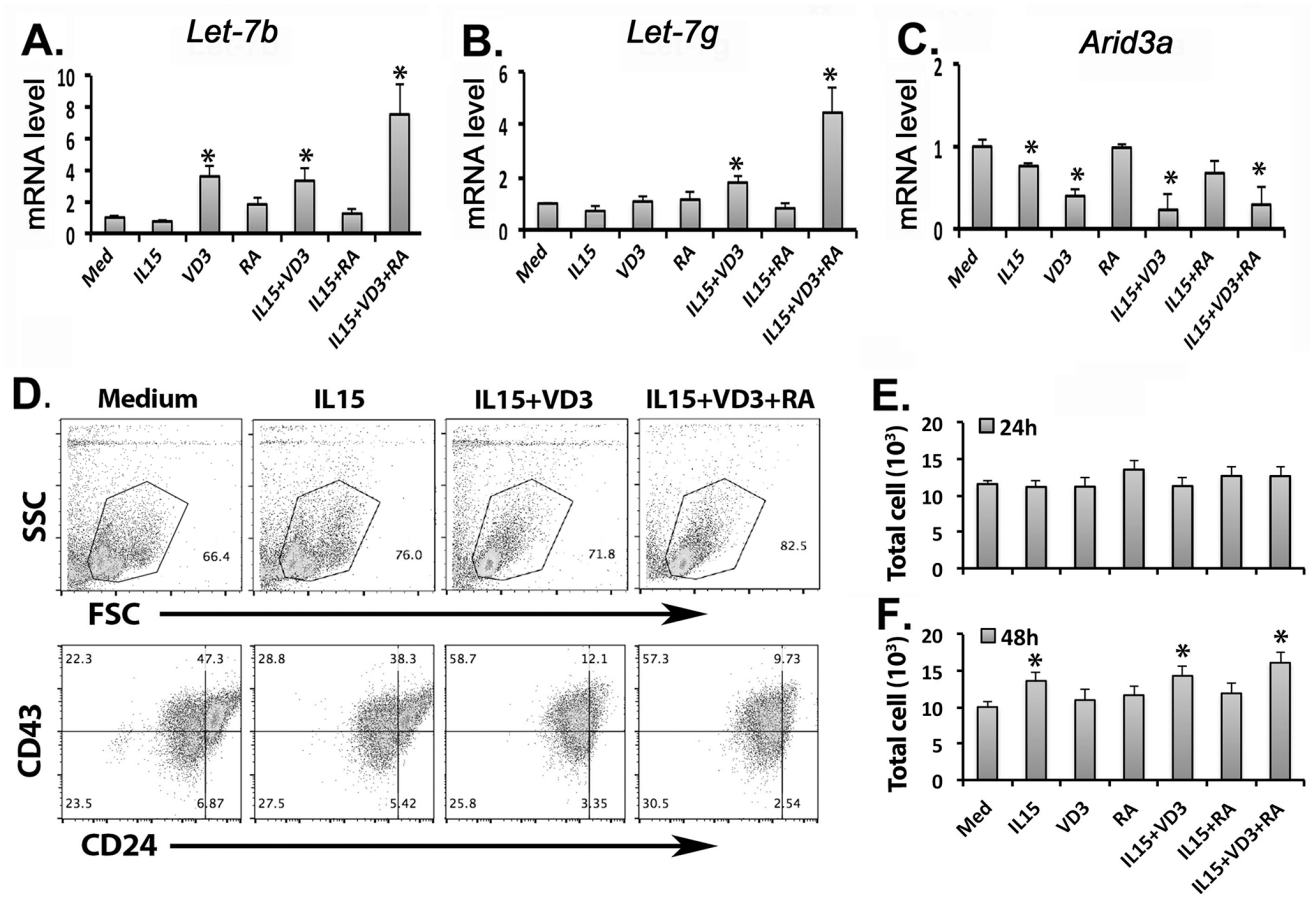
The effects of *Let-7* up-regulation signals on the development of thymic B progenitors. **(A-C)**. The thymic B progenitors (CD19^+^B220^+^CD24^+^CD43^+/lo^IgM^-^) sorted from around day 28 Z/N mice were co-cultured with IL15, VD3, and RA alone or various combinations. Gene expressions of *Let-7b* (A), *Let-7g* (B) and *Arid3a* (C) in cultured B cells were analyzed 48 hours after co-culture. **(D)**. The profiles of SSC and FSC (top), CD43 and CD24 staining (bottom) after co-culture with a medium, IL-15, IL-15+VD3 and IL-15+VD3+RA for 24 hours. **(E-F)**. The total cell numbers harvested from each group after co-culture for 24 (E) and 48 (F) hours were shown. Data are representative of three independent experiments. Student’s t-test analysis between medium and various test group. *P <0.05, **P <0.01, ***P <0.001. Bars indicate means ± SEM.

To test whether replacing these microenvironment-derived signals is sufficient to up-regulate *Let-7*, we sorted the major thymic B progenitor population (CD19^+^B220^+^CD24^+^CD43^+/lo^IgM^-^) from *Foxn1*^*lacZ*^ mutant thymus, and then co-cultured with *Cyp27b1*, *Aldh1a2* and *IL15* individually or in combination *in vitro*. After 48h, VD3 alone caused a 3.6-fold increase of *Let-7b* but no significant change in *Let-7g*, while IL15 or RA alone did not show significant effect on these two *Let-7s*. However, the combination of all three compounds increased both *Let-7b* (7.5-fold) and *Let-7g* (4.5-fold) expression (Fig 6A and 6B). Consistent with the increase in *Let-7s*, significant reductions of *Arid3a* expression were seen after treatment with VD3, IL15+VD3, and IL15+VD3+RA (Fig 6C). Phenotypic analysis showed that all CD24^hi^ large size B progenitors differentiated into CD24^+^ small size cells after stimulation with IL15+VD3 or IL15+VD3+RA at 24 hrs after co-culture (Fig 6D). The total numbers of cells had no significant change in all groups at 24 hrs (Fig 6E) but were significantly increased in IL15 groups at 48hr (Fig 6F) compared to the medium only control. These results indicated that the VD3 signaling pathway was the critical factor for up-regulation of *Let-7* in thymic B cells and that IL15 and RA had a synergistic effect with VD3. Thus, the deficiency of these signals prevented up-regulation of *Let-7*, and consequently increased the level of *Arid3a* in thymic B progenitors in the *Foxn1*^*lacz*^ thymus.

## Discussion

The requirement for the thymic microenvironment in specifying T lineage commitment and thymocyte differentiation is well documented; however, its role in the development of B cells in the thymus is less well understood. Although it has been long known that B cells are present and can develop within the thymus [33–36], it is only recently that any *in vivo* evidence for functional significance has been identified [37,38]. Our data provide evidence for a critical role for the thymic microenvironment and in particular for TECs in providing signals required for B cell development, proliferation, and differentiation in the thymus. Our data show that TECs produce a variety of factors and signaling molecules that regulate multiple stages of thymic B cell development, including the pathways that both influence lineage specification and control the balance of proliferation and differentiation in thymic B cell progenitors. Specifically, we provide evidence that *Let-7* up-regulation in the thymic B progenitors normally limits the generation of thymic B cells through the inhibition of Arid3a, and that this up-regulation requires FOXN1-dependent signals from the thymic epithelium.

We have shown in a related study that a wave of DN1ab thymic seeding progenitors present at postnatal day 7 in the thymus represents a wave of fetal derived hematopoietic cells seeding into the thymus (manuscript submitted to PLoS One). In the *Foxn1*^*lacz*^ thymus, reduced levels of FOXN1 cause a defective thymic microenvironment that modifies the fates and development of cells from this wave. The result is a reduction in TCRαβ progenitors (DN1a,b) paired with a transient amplification of progenitor B cell production. This study supported previous work showing that the intrinsic B potential of HSCs changes during the neonatal to adult transition [16,17], but that the microenvironment must control the expression of this intrinsic B cell potential. The current study builds on this result, to investigate the cell-intrinsic and microenvironmental signals that regulate thymic B cells production during the switch of HSCs from fetal to adult-type [3–5,39]. Our data showed that up-regulation of *Let-7* in thymic B progenitors normally suppresses B-1a fetal type B cell production by suppression of *Arid3a* expression, specifically by suppressing proliferation of B progenitors and promoting the differentiation from pre-B II cells to IgM^+^ immature B cells in *the Foxn1*^*lacz*^ mutant thymus. In addition, the repressive effect of a relative high level of *Let-7* seems only essential for the thymocyte development during neonatal to young adult when the TSPs posses a high B lineage potential. After adult-type HSCs seed the thymus, TECs down-regulate the signals that promote *Let-7* expression, resulting in their decline in intrathymic B progenitors. Thus, this mechanism appears to be specifically tuned to promote T cell production from fetal-derived HSCs during the perinatal period.

We also showed that *Lin28* is down regulated with normal timing after day 14 in both controls and Z/Z mutants, but that *Let-7* was not properly up-regulated at this time in the Z/Z mutants (Fig 2B), indicating that *Let-7* up-regulation is controlled by additional mechanisms other than down-regulation of *Lin28*. Recent work demonstrated that *Let-7* in hematopoietic cells regulates the terminal differentiation of NKT cells in the thymus, and is critically mediated by signals derived from the thymic microenvironment [14]. Indeed, our data show that the same signals from mTECs, VD3, RA, and IL15, were required for *Let-7* up-regulation and *Arid3a* down-regulation in thymic B progenitors as well. These results indicate that by controlling up-regulation of *Let-7*, the thymic microenvironment plays an important role not only specifically for B lineage fate, development, and differentiation but also more broadly for regulating the neonatal to the adult transition of lymphoid progenitors in the thymus.

Interestingly, we noticed that expression of *Let-7* dropped quickly after day 28 (Fig 1D and 1E), consistent with dynamic reduction of NKT and thymic B cells at this time period (Fig 5C and 5D), and with the known timing of HSCs switching from fetal to adult type, which is completed by postnatal 4 weeks [3–5]. Thus, the thymic B progenitors before 28 days should represent primarily fetal derived TSPs, while those after that time point are adult type. This decline in *Let-7* expression also coincides with declines in all three TEC-derived factors that promote their expression (Fig 4G–4I), which likely explains this decline. What is less clear is why the expression of these factors decreases between 4 and 6 weeks of age, and whether this is due to a TEC-intrinsic property, cross-talk with changing hematopoietic-derived populations (fetal vs adult), or physiological factors.

*Arid3a* expression also declined between 28 and 42 days (Fig 1F), which seems to conflict with our conclusion that the up-regulation of *Let-7s* control thymic B cells by down-regulating Arid3a expression in thymic B progenitors. *Arid3a* is required for normal early B lineage development and maintaining the fetal type profile [11,13], thus, the down-regulation of *Arid3a* after 28 days could be the molecular mechanism for the low B potential of adult type TSPs. The question remains, why is *Arid3a* down regulated at this time, when *Let-7* levels are also declining? As *Let*-7 levels do not go all the way down to their neonatal baseline, it is possible that the low level of *Let-7* that remains is sufficient to repress *Arid3a* expression. Alternatively, some other negative regulator of *Arid3a* expression could be present in adult-type HSCs.

## Conclusion

Taken together, our data show that the thymic microenvironment, especially MHCII^hi^ mTECs, provides necessary signals to up-regulate *Let-7* in the fetal type B progenitors in the neonatal thymus, thus controlling the development of thymic B progenitors specifically in the perinatal period, via down-regulation of *Arid3a*. This function is a critical component of regulating the balance of thymic production of T and B cells during the switch from fetal to adult type progenitors during the neonatal to adult transition. However, this high level of *Let-7* is not required to suppress thymic B cell production from adult-type BM derived TSPs. Given the broad regulatory effects of *Lin28/Let-7*, and the role of thymic B cells in central tolerance, our findings have potential implications for improving the transplantation of umbilical cord blood cells or adult BM cells, autoimmune disease, and for understanding the contributions of microenvironmental signals in cancer formation due to the disorder of *Lin28/Let-7* axis [40].

## Supporting information

S1 Fig Comparison of thymic B cells in Wt and +/Z mice. BL6 Wt mice were crossed with +/Z mice to generate Wt and +/Z mice. Total thymocytes were analyzed at age of 6–9 weeks. **(A)**. Total thymocytes from Wt and +/Z mice were gated on DN cells (left panels), and then gated on the DN1 subset (middle panel), the thymic B cells profiles of CD19 and B220 were shown on DN1 subset (right panel). **(B)**. The summary data of the percentage of thymic B cell in Wt and +/Z mice.
(TIF)

S1 File NC3Rs ARRIVE guidelines checklist thyB2.pdf. (PDF)
