# Supplementary figures and images for: Thymic epithelial cell-derived signals control B progenitor formation and proliferation in the thymus by regulating Let-7 and Arid3a
Source: PLoS One. 2018 Feb 20;13(2):e0193188. doi: 10.1371/journal.pone.0193188 (PMC5819816; doi:10.1371/journal.pone.0193188)

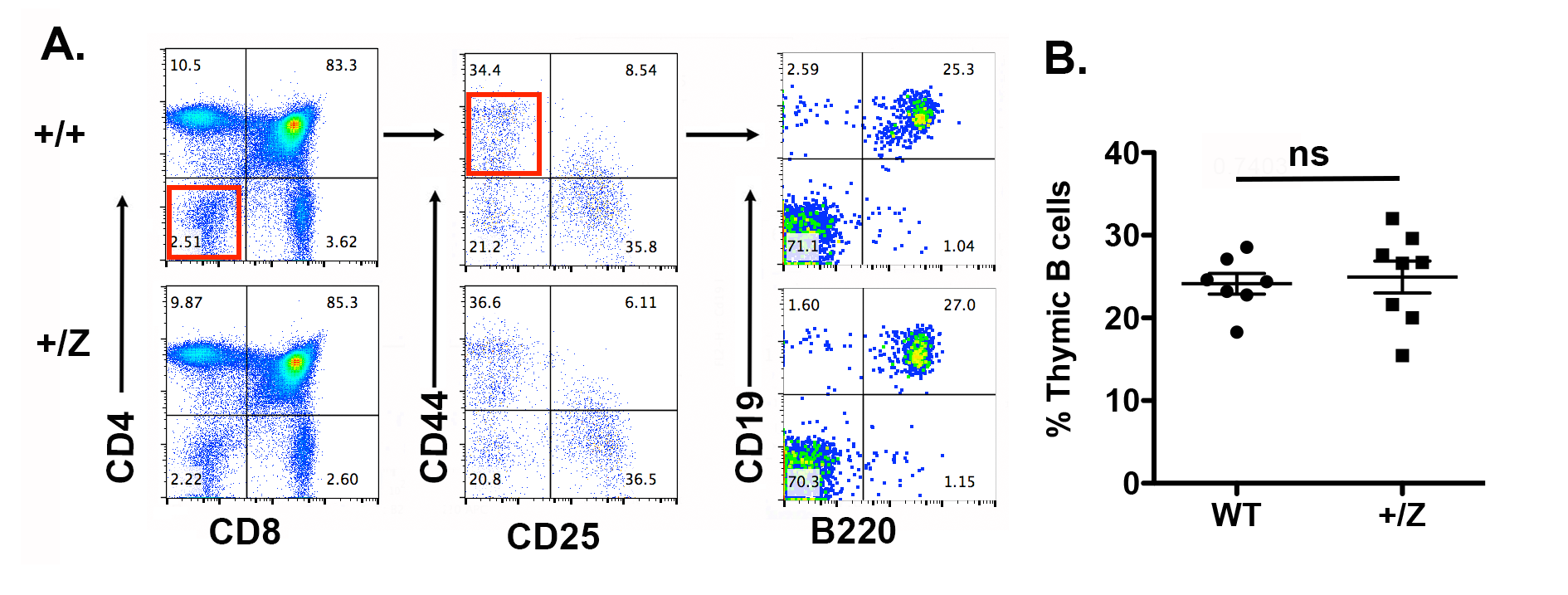

Supplement: S1 Fig — BL6 Wt mice were crossed with +/Z mice to generate Wt and +/Z mice. Total thymocytes were analyzed at age of 6–9 weeks. (A). Total thymocytes from Wt and +/Z mice were gated on DN cells (left panels), and then gated on the DN1 subset (middle panel), the thymic B cells profiles of CD19 and B220 were shown on DN1 subset (right panel). (B). The summary data of the percentage of thymic B cell in Wt and +/Z mice. (TIF) [file pone.0193188.s001.tif]
